# Examination of Inertial Sensor-Based Estimation Methods of Lower Limb Joint Moments and Ground Reaction Force: Results for Squat and Sit-to-Stand Movements in the Sagittal Plane

**DOI:** 10.3390/s16081209

**Published:** 2016-08-01

**Authors:** Jun Kodama, Takashi Watanabe

**Affiliations:** 1 Graduate School of Engineering, Tohoku University, Sendai, Miyagi 980-8579, Japan; jun.kodama@bme.tohoku.ac.jp; 2 Graduate School of Biomedical Engineering, Tohoku University, Sendai, Miyagi 980-8579, Japan

**Keywords:** joint moment, inertial sensor, trunk model, ground reaction force, center of pressure

## Abstract

Joint moment estimation by a camera-based motion measurement system and a force plate has a limitation of measurement environment and is costly. The purpose of this paper is to evaluate quantitatively inertial sensor-based joint moment estimation methods with five-link, four-link and three-link rigid body models using different trunk segmented models. Joint moments, ground reaction forces (GRF) and center of pressure (CoP) were estimated for squat and sit-to-stand movements in the sagittal plane measured with six healthy subjects. The five-link model and the four-link model that the trunk was divided at the highest point of the iliac crest (four-link-IC model) were appropriate for joint moment estimation with inertial sensors, which showed average RMS values of about 0.1 Nm/kg for all lower limb joints and average correlation coefficients of about 0.98 for hip and knee joints and about 0.80 for ankle joint. Average root mean square (RMS) errors of horizontal and vertical GRFs and CoP were about 10 N, 15 N and 2 cm, respectively. Inertial sensor-based method was suggested to be an option for estimating joint moments of the trunk segments. Inertial sensors were also shown to be useful for the bottom-up estimation method using measured GRFs, in which average RMS values and average correlation coefficients were about 0.06 Nm/kg and larger than about 0.98 for all joints.

## 1. Introduction

Elderly persons with decreased muscle strength and motor disabled subjects have some difficulties in their daily life. Especially, lower motor functions such as sit-to-stand and walking are important for independent daily life. Therefore, those subjects need rehabilitation training to improve or restore their motor functions. For the rehabilitation training, evaluation of motor function of the subjects is necessary to determine rehabilitation program. Recently, inertial sensors have been used to evaluate motor function: assessment of lower motor function for knee osteoarthritis patients [1], balance and knee extensibility evaluation of hemiplegic gait [2], effect of robotic gait rehabilitation [3], effect of drop foot correction by FES [4], gait analysis for outcome measurement after knee arthroplasty [5], and assessment of daily-life reaching performance after stroke [6]. Most of the studies using inertial sensors measure movement time, calculate joint or inclination angles, walking speed, step or stride length and segment position relative to other position, and detect gait event timings.

Joint moments also make it possible to evaluate muscle strength, training effect of rehabilitation and so on. Especially, lower limb joint moments have been studied for evaluation of muscle strength in relation to balance recovery performance [7,8]; movement strategies [9,10] of elderly subjects, and in evaluation of individuals with Parkinson's disease [11], hemiparesis [12] and cerebral palsy [13,14]; and used as evaluation indexes for rehabilitation [15,16,17], joint arthroplasty [18,19,20] and tuning of ankle-foot orthoses [21,22]. These various studies show that joint moments can be useful as one of quantitative indexes of rehabilitation. However, joint moments cannot be measured directly during movements in daily living. Generally, joint moments are estimated by an indirect way from data measured with a camera based 3D motion measurement system and a force plate system using a bottom-up inverse dynamics analysis [23]. Since the moment estimation method using these systems (conventional method) limits measurement environment and is costly, the estimation of joint moments is limited to the use in laboratories and major hospitals.

In order to eliminate the limitations in the conventional joint moment estimation method, a wearable system consisting of inertial sensors and force sensors has been studied [24,25]. In addition, estimates of back load using top-down inverse dynamics analysis for data measured with 17 inertial sensors for 16 body segments has been found to closely correspond to laboratory-based systems [26]. The sensor-based method can remove the limitation of the conventional method and reduce its cost for the measurement systems, and the joint moment estimation is considered to be easier to use by decreasing the number of sensors. Since moment estimation method only using inertial sensors uses a top-down inverse dynamics analysis [27,28], modeling of multi-joint segments of the trunk is an important issue for reliability and practicality of estimation of lower limb joint moments with inertial sensors. As Zijlstra et al. reported that a segmented trunk model yielded better results in estimation of hip abduction moment than a rigid trunk model [29], a segmented trunk model is considered to be effective for estimation of lower limb joint moments rather than a rigid trunk model.

This paper aims to evaluate quantitatively inertial sensor-based estimation methods of joint moments and ground reaction forces using five-link, four-link and three-link rigid body models that consist of different trunk models (three-segmented trunk model, two-segmented model and a rigid trunk model). Especially, lower limb joint moments in the sagittal plane were the focus, and sit-to-stand and squat movements were measured, because lower limb joint moments in the sagittal plane have been used in various studies, as mentioned above, and such movements were considered to be suitable for evaluating trunk segmented models. Lower limb joint moments and ground reaction forces (GRF) during squat and sit-to-stand movements were estimated with two methods using inertial sensors, and compared to those estimated by the conventional method using camera-based 3D motion measurement system and a force plate. The joint moments were also estimated by a camera-based estimation method without a force plate in order to evaluate difference between the top-down and the bottom-up methods. Then, estimation methods of joint moments and ground reaction forces using inertial sensors were discussed considering easiness of setting of the measurement devices.

## 2. Materials and Methods

### 2.1. Rigid Body Link Model

**Figure 1:**
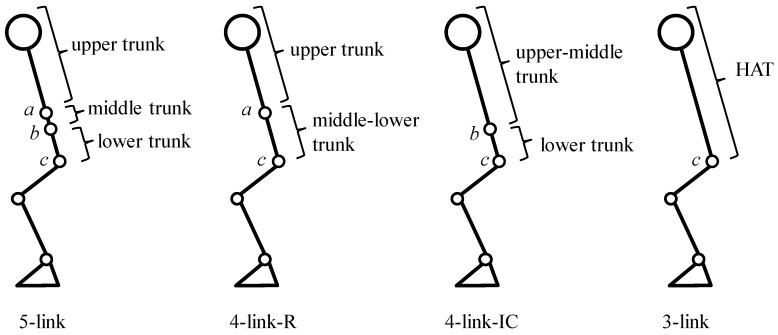
Multi-link models tested in this paper. *a*, *b*, and *c* show the lower end of the rib, the highest point of the iliac crest and the great trochanter, respectively.

Human body was represented by the rigid body link model and assumed that each segment moves in the sagittal plane symmetrically and the feet were fixed on the ground. Figure 1 shows rigid body link models tested in this paper. Based on the five-link model, in which the trunk was divided into three segments based on Young et al. [30], three other types of trunk models were constructed: two-segmented trunk model divided at the lower end of the rib (four-link-R model), two-segmented trunk model divided at the highest point of the iliac crest (four-link-IC model) and a rigid trunk model (three-link model). The head and the arm segments were included in the top segment of each link model.

Body segment parameters (i.e., segment length and mass, center of mass position and moment of inertia) were calculated based on the inertial properties of body segments of young Japanese athletes as reported by Ae et al. [31]. Since Ae et al. used a model that divided the trunk into upper and lower parts at the lower end of the rib, values of body segment parameters of the four-link-R model were calculated first. Then, those values were converted to those of the five-link model by modeling the middle trunk as an elliptic cylinder with uniform density based on the method of calculating body segment parameters of the total system [23], in which the value of the density reported by Clauser et al. [32] and size parameters obtained from the Advanced Industrial Science and Technology (AIST) Human body size database [33] were used. After that, body segment parameters of the four-link-IC and three-link models were calculated from those of the five-link model and the four-link-R model, respectively, by combining two segments [23]. That is, the upper and the middle trunk segments of the five-link model were combined into the upper-middle trunk of the four-link-IC model and the upper and the middle-lower trunk segments of the four-link-R model were combined into the head, arm and trunk (HAT) segment of the three-link model. The calculated values of body segment parameters are shown in Table 1. Here, foot segment was model by a triangle and height of the center of mass (CoM) of foot segment was assumed to be 3 cm.

### 2.2. Estimation of Joint Moment

**Figure 2:**
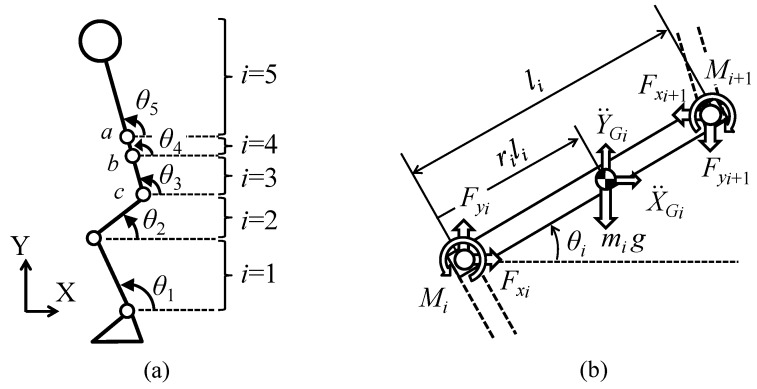
Definitions of segment inclination angle, joint reaction force and joint moment: (**a**) inclination angle of the five-link model, where *a*, *b* and *c* are landmarks of the body shown in Figure 1; and (**b**) joint reaction forces and joint moments at segment *i*.

Joint moments were estimated by solving equations of translational motion and rotational motion of the CoM of each segment. Figure 2 shows definitions of inclination angles, joint moments and joint reaction forces for the five-link model of human body. The origin of the coordinate system was set at ankle position, in which heel position was 3.1 (%height) and −4.2 (%height).

The five-link model consists of the foot, the shank (*i* = 1), the thigh (*i* = 2), the lower trunk (*i* = 3), the middle trunk (*i* = 4) and the upper trunk (*i* = 5). The segment numbers were assigned from the bottom (shank segment) to the top segments. The motion equations for segment *i* are represented by the following (Figure 2b):

(1) $$m_{i}{\ddot{X}}_{G_{i}}=F_{x_{i}}-F_{x_{i+1}}$$

(2) $$m_{i}{\ddot{Y}}_{G_{i}}=F_{y_{i}}-F_{y_{i+1}}-m_{i}g$$

(3) $$I_{i}{\ddot{\theta }}_{i}=M_{i}-M_{i+1}+\left(r_{i}F_{x_{i}}+\left(1-r_{i}\right)F_{x_{i+1}}\right)l_{i}\sin\;\theta_{i}-\left(r_{i}F_{y_{i}}+\left(1-r_{i}\right)F_{y_{i+1}}\right)l_{i}\cos\;\theta_{i}$$

where i shows segment number; $m_{i}$, $l_{i}$, $r_{i}$ and $I_{i}$ are body segment parameters (segment mass, segment length, CoM position ratio from the distal end and moment of inertia, respectively); $M_{i}$ and $F_{x_{i}}$ and $F_{y_{i}}$ are joint moment and joint reaction forces, respectively; ${\ddot{X}}_{G_{i}}$ and ${\ddot{Y}}_{G_{i}}$ are accelerations of segment CoM position in the horizontal and the vertical direction, respectively; $\theta_{i}$ and ${\ddot{\theta }}_{i}$ are inclination angle and its angular acceleration, respectively; and g shows gravitational acceleration. Estimated joint moments were obtained by substituting joint reaction forces calculated from Equations (1) and (2) into Equation (3). For each subject, values of body segment parameters were calculated using measured height and weight and inclination angles were measured during movements. Accelerations of the inclination angles and segment CoM positions were calculated using calculated body segment parameters and measured inclination angles.

Joint moments were estimated using the following four methods:

(1) Conventional method (bottom-up method): The conventional method used a 3D motion measurement system and a force plate. First, segment inclination angles were calculated based on position data of markers attached on landmarks of the body. Then, segment CoM positions were calculated from the bottom segment to the top segment using the inclination angles and ratios of segment length and center of mass position shown in Table 1. Segment length was calculated from height of subject. CoM accelerations and angular acceleration of each segment were calculated by the differential with a 3rd order low-pass differential algorithm proposed by Usui et al. [34]. Here, a zero-phase 2nd order low pass Butterworth filter with a cutoff frequency of 6 Hz [35] was applied to the estimated angles before calculating the differentials because the low-pass differential algorithm could not reduce sufficiently high frequency noise in CoM accelerations caused by calculation of the 2nd-order differential of CoM position. Joint moments were estimated by solving simultaneous Equations (1)–(3) using the calculated values and measured ground reaction forces from the bottom joint to the top joint. That is, ankle joint moment was estimated first, and then, knee joint moment is estimated using the ankle joint moment and joint reaction forces.

(2) SI method (Sensor-measured Inclination angle-based method): The SI method only used inertial sensors. Joint moments were estimated from the top joint to the bottom joint using inclination angles with the assumption that no external force acts on the top segment (top-down method). Segment inclination angles were estimated from signals measured with inertial sensors by the integral of angular velocities using Kalman filter [36]. The Kalman filter was used for correcting integration error using acceleration signals measured with the sensors, in which the zero-phase 2nd order Butterworth low pass filter with a cutoff frequency of 1 Hz was applied to the acceleration signals. Then, the inclination angles were low-pass filtered by the zero-phase 2nd order Butterworth filter with a cutoff frequency of 6 Hz for the same reason as the conventional method. After that, CoM positions, CoM accelerations and angular accelerations of all segments were calculated by the same way as the conventional method using the estimated angles. Finally, joint moments were estimated by solving the simultaneous Equations (1)–(3) substituting those calculated values.

(3) CI method (Camera-measured Inclination angle-based method [27,28]): The CI method used only a camera-based motion measurement system. Inclination angles, CoM positions, CoM accelerations and angular accelerations of all segments were calculated by the same way as the conventional method. Joint moments were estimated by the top-down method used in the SI method.

(4) SA method (Sensor-measured Acceleration-based method): The SA method used only inertial sensors. Joint moments were estimated by the top-down method using acceleration signals measured with inertial sensors in addition to estimated segment inclination angles, which was modified as a sensor-based method from the method by Zijlstra et al. [29] because they used trunk angle calculated based on position data of markers. First, segment inclination angles were estimated by the same way as the SI method using Kalman filter and the low pass filter with a cutoff frequency of 6 Hz. Angular accelerations were calculated by the differential of measured angular velocities using the 3rd order low-pass differential algorithm. Accelerations of each segment CoM were calculated from measured accelerations with the inertial sensor attached on the CoM of its segment, in which measured acceleration signals were converted to values in the coordinate system shown in Figure 2a by the rotation matrix calculated by the estimated angles, and gravitational acceleration were removed. Here, CoM accelerations of combined segments such as the HAT segment of three-link model were calculated from acceleration signals of two inertial sensors. Joint moments were estimated by the same way as the SI method from the top to the bottom joints.

### 2.3. Experimental Method

**Figure 3:**
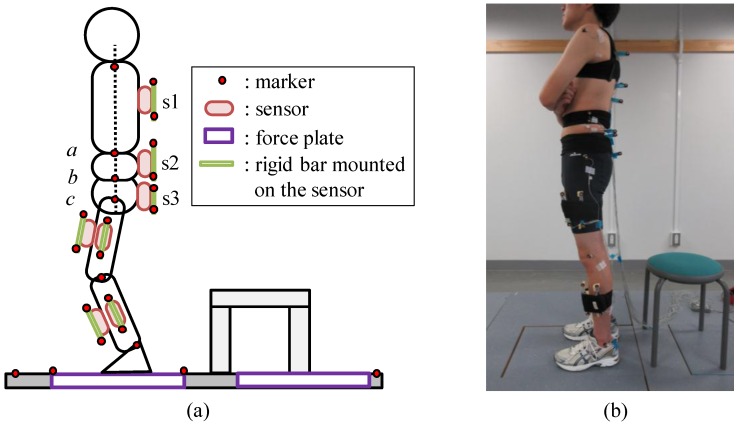
Experimental setup of measurement of movements with inertial sensors, 3D motion measurement system and force plates: (**a**) attachment positions of inertial sensors and markers of the 3D motion measurement system and placement of force plates; and (**b**) a picture of a subject with attached markers and sensors.

Six young healthy subjects (male, 21–23 years old, 176.3 ± 4.0 cm, 63.9 ± 6.4 kg) performed four types of movements five times: two types of squat movement (movement time was 6 s and 9 s) and two types of sit-to-stand movement (normal condition and condition of forward inclination of the trunk simulating elderly persons). The movements were measured with the camera-based 3D motion measurement system (OPTOTRAK, Northern Digital), two force plates (9286A, Kistler) and wireless inertial sensors (custom-made sensor modules using Bluetooth and InvenSense MPU-9150) at a sampling frequency of 100 Hz. Figure 3 shows the experimental set up. Inertial sensors were attached to the skin or on half tights (BIOGEAR A60BP, MIZUNO Corporation, Tokyo, Japan) with double-sided adhesive tape and stretch bands near the CoM positions of body segments corresponding to those of the five-link model as shown in Figure 3a. Seven inertial sensors were mounted on the upper, middle and lower trunk segments, and on the frontal and the lateral sides of the shank and the thigh of the left lower limb. Markers for the 3D motion measurement system were attached on the left side of the subject, in which attached positions were the acromion, along the long axis of the trunk at the same height as the lower end of the rib and the highest point of the iliac crest, the great trochanter, the lateral femoral condyle, and the lateral malleolus. In addition, a rigid bar on which a pair of markers was attached was mounted on each inertial sensor in order to validate inclination angles estimated by signals measured with the sensors, although the angle estimation method has been shown to estimate inclination angles of lower limbs with average root mean square (RMS) error less than 4° for human gait movements and cyclic movements of rigid bodies [36,37]. Force plates were used for measurement of ground reaction forces and load acting on a stool that was used to detect the seat-off timing.

Subjects were instructed to perform movements with their arms folded, as seen in Figure 3b. The sound of metronome was presented to the subjects for regulating movement speed. Measured data were evaluated in estimation of segment angles first. Here, both of angles measured with the 3D motion measurement system and estimated with inertial sensors were calibrated using angles during quiet standing for 0.5 s. Significant differences were evaluated using t-test with Bonferroni correction.

## 3. Results

Estimated inclination angles with the inertial sensors and estimated joint moments with the SI, CI and SA methods were evaluated for movement period. The movement period was detected by angular velocity of the shank and the thigh for squat movement and by ground reaction force of the stool for sit-to-stand movement, respectively.

### 3.1. Estimation of Inclination Angles

**Figure 4:**
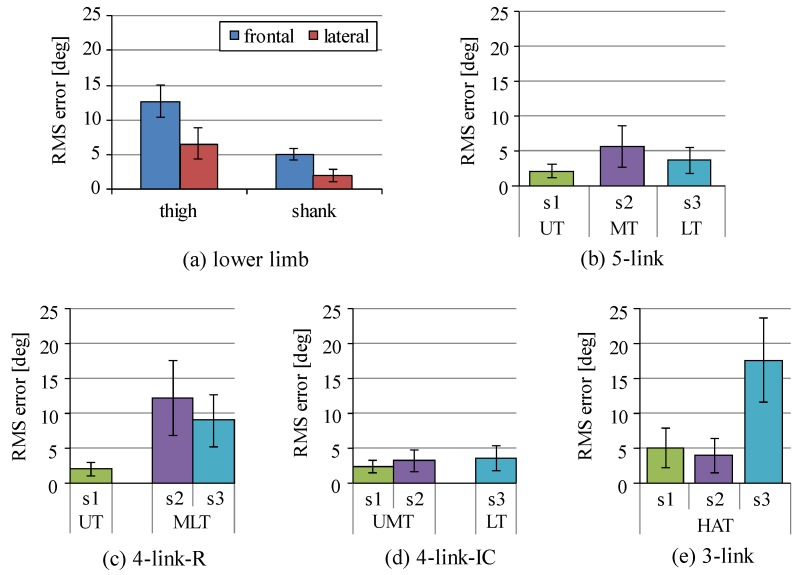
Root mean square (RMS) errors of segment inclination angles between estimation with inertial sensors and measurement with markers attached on the landmarks. s1, s2 and s3 show sensors mounted on the trunk as shown in Figure 3a. (**a**) RMS errors calculated for the thigh and the shank segments; (**b**) RMS errors for the upper trunk (UT), the middle trunk (MT) and the lower trunk (LT) of the five-link model; (**c**) RMS errors for the upper trunk (UT) and the middle-lower trunk (MLT) of the four-link-R model; (**d**) RMS errors for the upper-middle trunk (UMT) and the lower trunk (LT) of the four-link-IC model; and (**e**) RMS errors for the trunk segment (HAT) of the three-link model.

Segment inclination angles estimated by signals measured with the inertial sensors were compared to those angles measured with 3D motion measurement system in order to determine sensor positions to estimate the angles for joint moment estimation. Figure 4 shows root mean square (RMS) errors of the inclination angles between estimation with the inertial sensors and measurement with markers attached on landmarks of the human body, which are averaged values of all trials for all four movements measured with all six subjects. For the thigh and the shank segments, the inertial sensors attached on the lateral side showed smaller values of RMS error than those of sensors on the frontal side. For the middle-lower trunk segment of the four-link-R model, sensor s3 mounted on the lower trunk showed smaller RMS error for the middle-lower trunk (MLT) than that of s2. The inclination angle of the upper-middle trunk (UMT) of the four-link-IC model was estimated with good accuracy using either sensor s1 or s2. For the HAT segment of the three-link model, sensor s2 mounted on the middle trunk showed the smallest RMS error.

**Figure 5:**
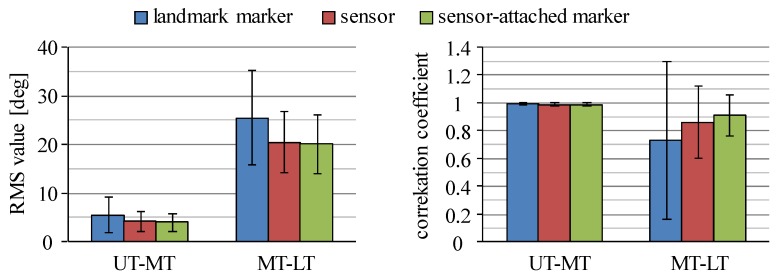
RMS values and correlation coefficients of inclination angles between the upper and the middle trunk segments (UT-MT) and between the middle and the lower trunk segments (MT-LT) of the five-link model. UT, MT and LT are the upper, the middle and the lower trunk segments, respectively.

As shown in Figure 4, RMS errors of inclination angle of the middle-lower trunk (MLT) were larger than other trunk segments (UT, MT, LT and UMT). Figure 5 shows the RMS values and correlation coefficients of inclination angles between the upper and the middle segments and between the middle and the lower segments of the five-link model. Movements of the upper and the middle segments of the trunk are shown to be similar during squat and sit-to-stand movements performed in this study, while there are large differences in movements between the middle and the lower segments. This result is not consistent between sensors and 3D motion measurement system.

The average values of RMS error and correlation coefficient of the inclination angles between the inertial sensors and the markers mounted on each sensor by the rigid bar are shown in Table 2. Average values of RMS error were less than 2.5° and correlation coefficients were larger than 0.98 showing almost 1.0 for all the segments. This shows that the angle estimation method used in this study is reliable, although the RMS errors of some segments were large, as shown in Figure 4. Therefore, sensor s2 was used for the HAT segment of the three-link model and s3 was used for the lower-middle trunk of the four-link-R model. For the upper-middle trunk segment of the four-link-IC model, both sensors s1 and s2 were used for estimation of joint moments, since both of them showed values of RMS error less than 3° on average.

### 3.2. Joint Moment Estimation

**Figure 6:**
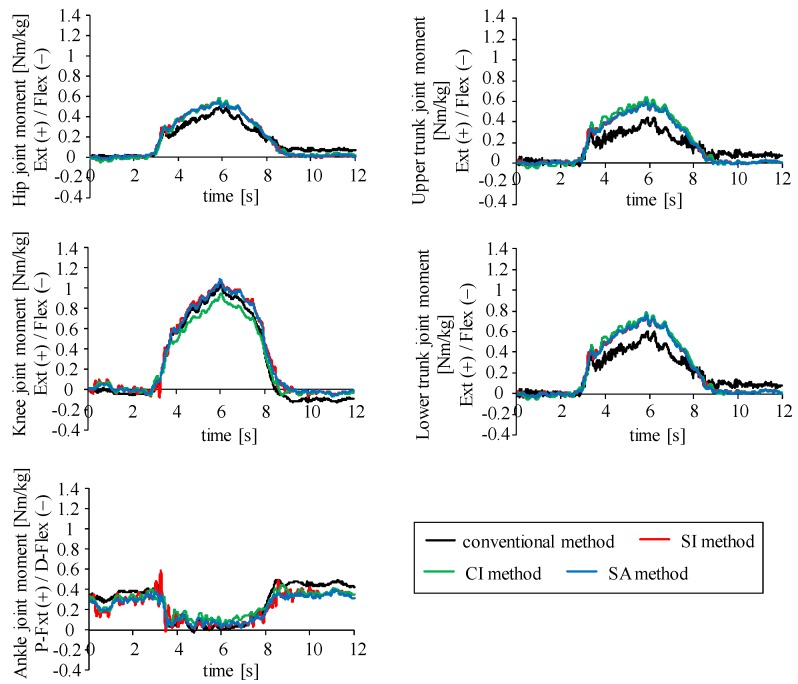
Examples of waveforms of estimated joint moments during squat movement (6 s) with four estimation methods (five-link-model). “Ext” and “Flex” show extension and flexion moments, respectively. “P-Flex” and “D-Flex” show plantar flexion and dorsiflexion moments of the ankle joint, respectively.

Using the inertial sensors that were determined in the previous section, joint moments were estimated with the four estimation methods. Figure 6 shows examples of waveforms of the joint moments estimated with five-link model for squat movement. All estimation methods showed similar waveforms.

**Figure 7:**
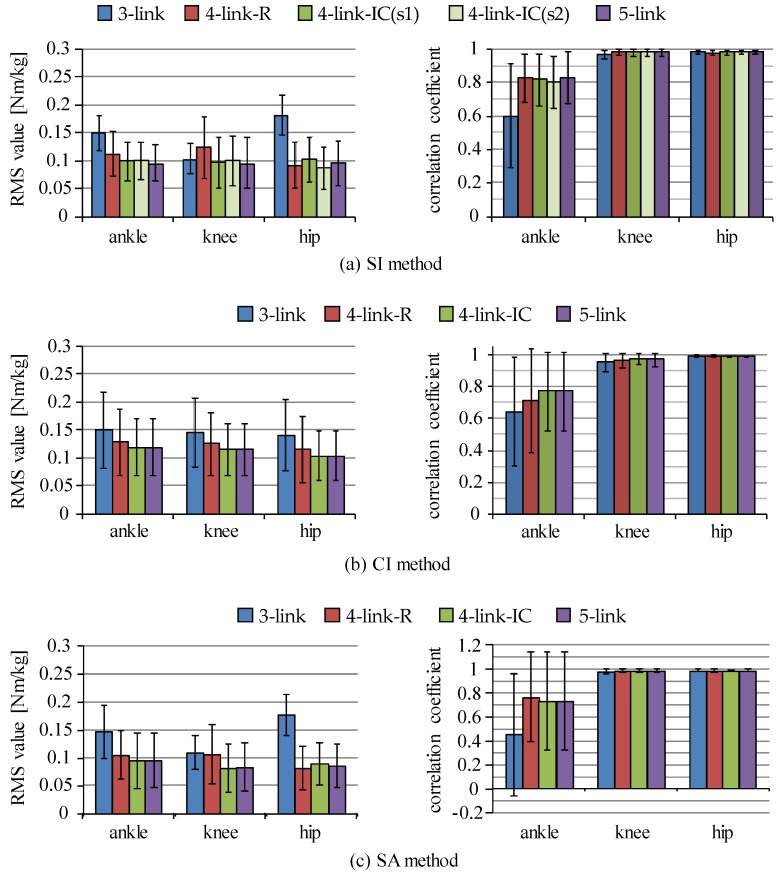
RMS values and correlation coefficients of estimated joint moments between the top-down methods and the conventional method: (**a**) SI method; (**b**) CI method; and (**c**) SA method. In the SI method, “(s1)” and “(s2)” for the four-link-IC model show the inertial sensor that was used for estimation of inclination angle of the upper-middle trunk to estimate joint moments.

**Figure 8:**
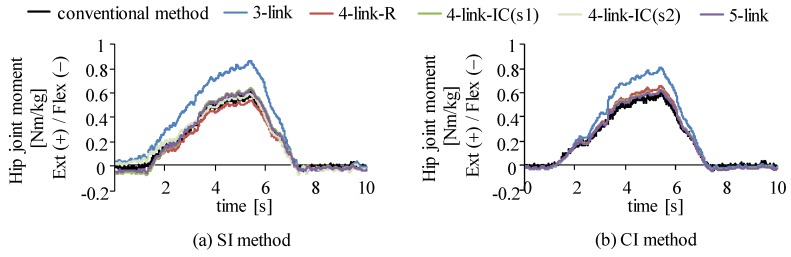
Examples of estimated hip joint moment in comparison between different trunk link models during squat movement (6 s): (**a**) estimation with the SI method and the conventional method; and (**b**) estimation with the CI method and the conventional method.

Four link models were compared in joint moment estimation for the SI, the CI and the SA methods. RMS values and correlation coefficients of estimated joint moments in comparison to those by the conventional method using a camera-based motion measurement system and a force plate are shown in Figure 7. Since body weights were different among subjects, joint moments were normalized by their body weights and those values obtained from all trials of all six subjects were averaged. For the SI method, joint moments were estimated with four-link-IC model using sensor s1 and s2, which are shown as four-link-IC (s1) and four-link-IC (s2) in the figure. All estimation methods showed that the five-link and the four-link-IC models estimated joint moments with smaller RMS values and larger values of correlation coefficient, in which the SI method showed RMS values of about 0.1 Nm/kg for all the lower limb joints, correlation coefficients of about 0.98 for the hip and the knee joints and about 0.80 for the ankle joint in average. There were no significant differences in the RMS and correlation coefficient values between the five-link and the four-link-IC models (p > 0.05). The three-link model was the worst, although RMS values for the knee joint moment were smaller than the ankle and the hip joints with the SI and the SA methods, respectively. The four-link-R model showed larger RMS values for knee joint moments than the five-link and four-link-IC models with the SI and the SA methods. Differences in the RMS and correlation coefficient values among the estimation methods were small when the five-link or four-link-IC models was used, although the SA method showed large variation of the correlation coefficients for the ankle joint moments. Figure 8 shows examples of waveforms of estimated hip joint moments with the SI and the CI methods in comparison to the rigid body link models. The three-link model overestimated the extension moments of the hip joint with both methods, which increased RMS values.

**Figure 9:**
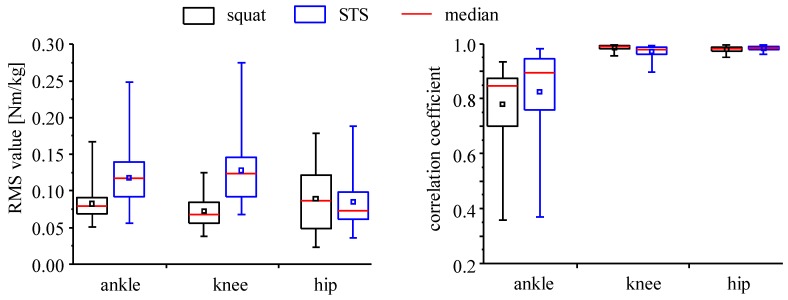
RMS values of joint moments between the conventional method and the SI method with the four-link-IC model using the sensor s2. “STS” shows the sit-to-stand movement. Red line shows the median and small square shows average value. The maximum and the minimum values are also shown.

Variations of RMS values shown in Figure 7 were relatively large. For example, standard deviations of the SI method with four-link models were about 0.04 Nm/kg, while average RMS values were about 0.1 Nm/kg. Since the RMS values shown in Figure 7 were averaged values of all the data obtained with six subjects for four types of movements, those standard deviations included variations among subjects, among movement conditions and among trials. Therefore, distributions of RMS values and correlation coefficients were calculated separately for squat movements and for sit-to-stand movements (Figure 9). The RMS values for sit-to-stand movements were larger than those for squat movements for knee and ankle joint moments (p < 0.01). Although there is no significant difference for the hip joint moment, variations for the squat movements were larger than the sit-to-stand movements. There was no significant difference in correlation coefficient for the ankle joint moment (p > 0.05). Although there were differences for knee and hip joint moments (p < 0.05), the minimum values of correlation coefficients were larger than about 0.9 for both movements.

**Figure 10:**
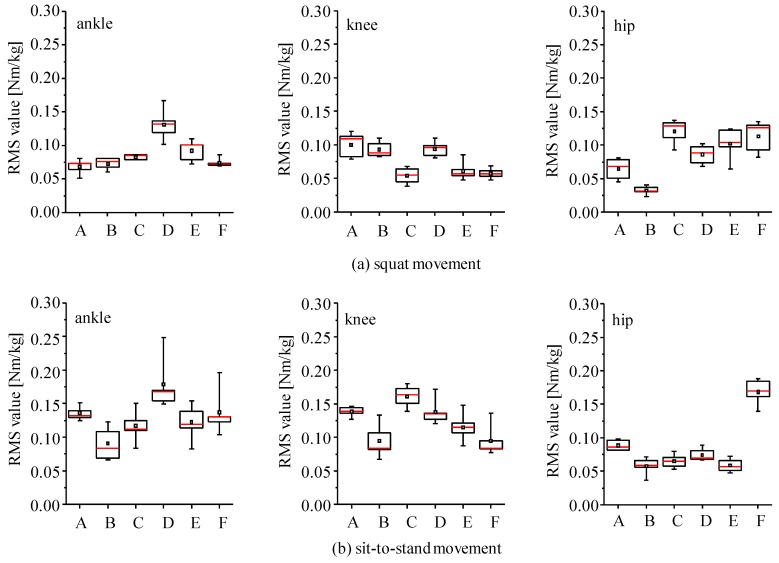
RMS values of joint moments between the conventional method and the SI method with the four-link-IC model using the sensor s2 for 6 Subjects. Red line shows the median and small square shows average value. The maximum and the minimum values are also shown: (**a**) squat movement (normal speed: 6 s); and (**b**) sit-to-stand movement (normal).

Then, RMS values of the six subjects were compared under the single movement condition (Figure 10). Differences of the median among the subjects were from 0.05 to 0.11 Nm/kg. On the other hand, ranges of distribution of RMS value between the 25th and 75th percentiles were from 0.003 to 0.04 Nm/kg. Most of the distribution range was less than 0.025 Nm/kg.

**Figure 11:**
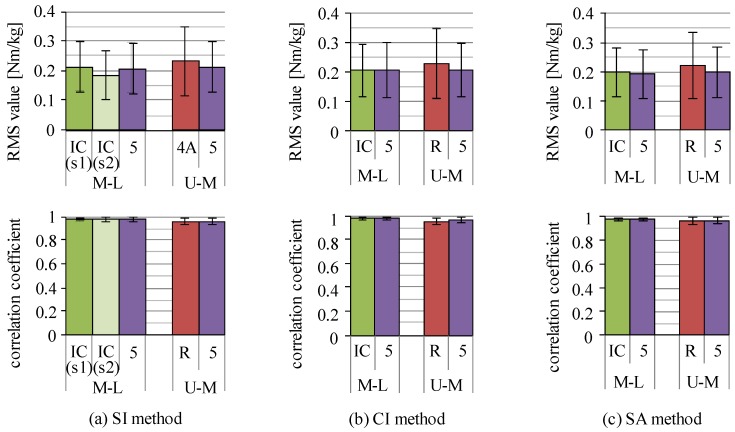
RMS values and correlation coefficients of estimated joint moments of the trunk link nodes: (**a**) SI method; (**b**) CI method; and (**c**) SA method. M-L and U-M are joints of the highest point of the iliac crest and the lower end of the rib, respectively. R, IC and 5 are the four-link-R, the four-link-IC and the five-link models, respectively. In the SI method, “(s1)” and “(s2)” are the same meaning as shown in Figure 7.

Except for the three-link model, joint moments of the link nodes of the trunk segment, that is, joint moments of the lower end of the rib and the highest point of the iliac crest, were estimated in addition to lower limb joint moments. Averaged RMS values and correlation coefficients of the joint moments of the link nodes of the trunk for each estimation method are shown in Figure 11. For all models and all estimation methods, RMS values were almost 0.2 Nm/kg and values of correlation coefficients were higher than 0.95. There were no large differences between link models and between estimation methods. 

**Figure 12:**
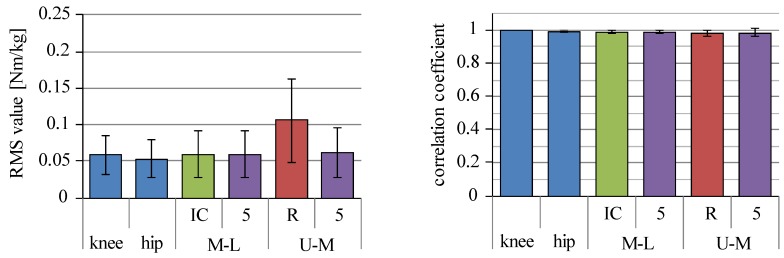
RMS values and correlation coefficients of joint moments between the conventional method and the bottom-up method using inertial sensors and force plate. M-L, U-M, IC, R and 5 are the same meaning as shown in Figure 11. Ankle joint moments are the same between both methods.

As reported by Faber et al., joint moments can be estimated with a camera-based motion measurement system and the shoes that measure ground reaction forces, which can remove partly the limitation of measurement environment of the conventional method [25]. Therefore, joint moments were estimated by the bottom-up method with inertial sensors and force plate. Figure 12 shows RMS values and correlation coefficients of the joint moments between the conventional method and the bottom-up estimation method with the inertial sensors and the force plate. Ankle joint moment estimated by the bottom-up method coincided with those by the conventional method. This result reflects influence of estimation errors of the inclination angles with the inertial sensors on joint moment estimation. Excluding the trunk link node of the four-link-R model, RMS values were about 0.06 Nm/kg. Correlation coefficients were larger than about 0.98 for all the joints.

### 3.3. Ground Reaction Force

**Figure 13:**
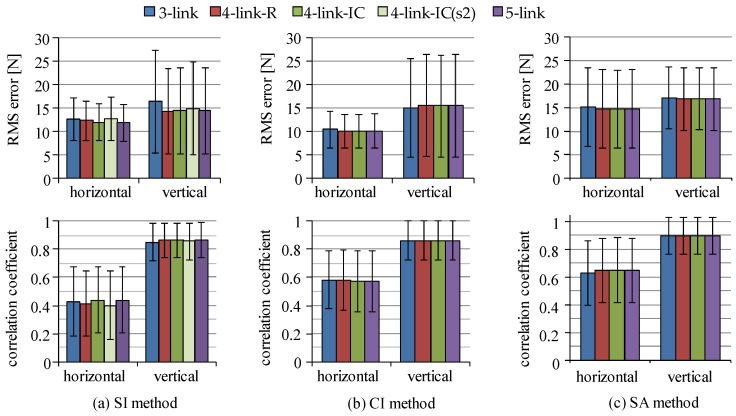
RMS errors and correlation coefficients of ground reaction forces between estimated values with each estimation method and those measured with the force plate; (**a**) SI method; (**b**) CI method; and (**c**) SA method. In the SI method, “four-link-IC” shows results with sensor s1.

**Figure 14:**
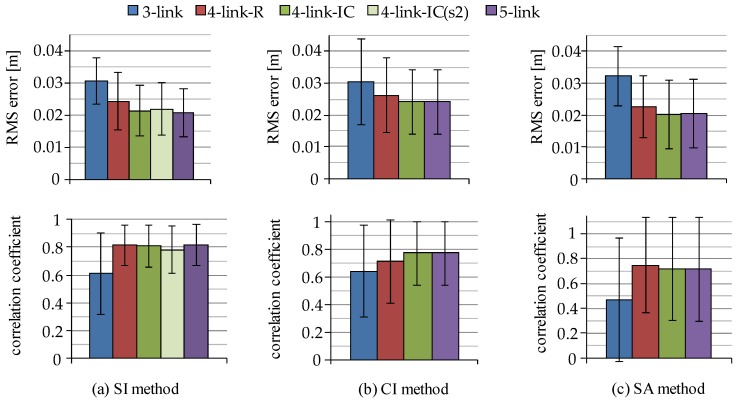
RMS errors and correlation coefficients of CoP between estimated values with each estimation method and those measured with the force plate; (**a**) SI method; (**b**) CI method; and (**c**) SA method. In the SI method, “four-link-IC” shows results with sensor s1.

Ground reaction forces and center of pressure (CoP) are generally measured with force plate. However, they can be estimated from the sum of products of acceleration of segment CoM position and its segment mass. The CoP is also calculated by substituting ground reaction forces in the horizontal and the vertical directions and ankle joint moment and reaction forces into the equation of rotational motion of the CoM of the foot segment. The ground reaction forces and the CoP were estimated with the SI, CI and SA methods, and the estimated values were compared to those measured with the force plate. Figure 13 and Figure 14 show RMS errors and correlation coefficients of the ground reaction forces and the CoP between the measured values and the estimated values. The errors and correlation coefficients of ground reaction forces in both directions were approximately constant regardless of the rigid body link model used for the estimation. On the other hand, those values of the CoP were improved using the five-link and the four-link-IC models for all the estimation methods.

## 4. Discussions

### 4.1. Estimation of Inclination Angles

For the shank and the thigh segments, RMS errors of inclination angles estimated with the lateral side sensors were smaller than those with the frontal side sensors in comparison to the markers attached on the landmarks of the body as shown in Figure 4. However, the RMS errors calculated with the angles measured with markers mounted on those sensors themselves were almost the same on both sides, and were less than 2.5°, as shown in Table 2. In our previous study, RMS errors of the lower limb inclination angles during gait movements between measurement with the 3D motion measurement system using landmark markers and estimation with the inertial sensors mounted on the frontal side were less than about 4° on average [36,37]. These show that the angle estimation method with the inertial sensors is reliable and has satisfactory estimation accuracy. Therefore, it is considered that movements of the body part where the sensor was attached were different between the frontal and the lateral sides and movements of sensor position were different from those of landmark positions during squat and sit-to-stand movements. Since the squat and sit-to-stand movements include larger flexions of the hip and the knee joints than gait movements, deformation of muscles caused by contact of the thigh and the shank or contact of the thigh and the stool before standing up, and movements of the skin where landmark markers attached on [38] are considered to increase RMS errors for squat and sit-to-stand movements. It is considered that the inertial sensor-based estimation of angles can be used for evaluation of movements and comparison to other data estimated with the same sensor-based method. A camera-based measurement system also has the problem that markers on the landmarks move with the skin during movements [38]. 

The four-link-IC model is suggested to be more appropriate to measure inclination angles of trunk segments than the four-link-R model. Results with the four-link-IC model shown in Figure 4 showed that both sensors, s1 and s2, could measure inclination angles of the upper-middle segment of the trunk with small RMS errors in comparison to those measured with landmark markers. The four-link-R model showed large RMS errors of inclination angles of the middle-lower segment of the trunk. As shown in Figure 5, the middle trunk moved differently from the lower trunk, while the upper-trunk moved similarly to the middle trunk. Trunk segmentation for four-link model is suggested to be better at the highest point of the iliac crest.

### 4.2. Estimation of Joint Moments and Ground Reaction Forces

All estimation methods showed that the five-link and the four-link-IC models estimated joint moments and CoP with smaller RMS values and larger values of correlation coefficient as shown in Figure 7 and Figure 14. There were no significant differences between the five-link and the four-link-IC models (p > 0.05). Segmented trunk models were shown to decrease differences in estimated joint moment between the top-down methods and the conventional method. This is the same as the results by Zijlstra et al., in which using a segmented trunk model was better for estimation of hip abduction moment than using a rigid trunk model [29]. On the other hand, as shown in Figure 9 and Figure 10, estimation accuracies were different between measured movements and among subjects. Further studies are required that decrease the variation among subjects and among movement conditions for the top-down methods. A possible cause of the variation among subjects is considered to be deviation of the body segment parameters of individual subject from their average values.

The overestimation of three-link model shown in Figure 8 is considered to be caused by the increase of moment arm of the trunk segment. The CoM position of the trunk segment of the three-link model is estimated as more forward than its actual position because the three-link model cannot represent bending of multi-segments of the trunk. The segmented trunk models with the top-down method are considered to decrease differences in joint moment estimation from the conventional method because bending of the trunk was expressed approximately.

RMS values of knee joint moments estimated by the SI and the SA methods with the three-link model (Figure 7a,c) were smaller than those values of hip joint moments. The overestimation of extension moment of the hip joint is considered to lead to underestimation of the knee joint extension moment with the top-down method. However, the inertial sensor mounted on the thigh segment estimated the inclination angle smaller than the camera-based 3D motion measurement system during bending of the trunk. Small inclination angle of the thigh segment leads to the increase of moment arm of the force of gravity and the joint reaction forces of the hip and knee joints. That is, decrease of knee Joint moment caused by overestimation of the hip joint moment is considered to be cancelled out by the underestimation of the inclination angle of the thigh segment. Therefore, it is considered that RMS values of knee joint moments for the SI and the SA methods with the three-link model were smaller than other joint moments.

As seen in Figure 8a, the maximum extension moment of the hip joint by the SI method using the four-link-R model was smaller than the maximum moment estimated by the conventional method, which was different from the CI method. A possible cause of the underestimate of the hip joint moment is that the inertial sensor mounted on the middle-lower trunk segment estimated inclination angle to be perpendicular to the horizontal plane more than the 3D motion measurement system. This causes underestimation of joint moment by decreasing moment arm of the force of gravity and the joint reaction forces for the middle-lower trunk segment. In addition, knee joint extension moment is underestimated by the overestimation of the hip joint extension moment. Therefore, in the case of using the four-link-R model with the SI method, the knee joint moment is considered to be underestimated, which caused increase of RMS values as shown in Figure 7.

Figure 13 and Figure 14 showed that the difference of the trunk models influenced the RMS values between measured and estimated CoP values, while estimation of ground reaction forces were not affected by the difference of trunk models. That is, segmented trunk models are shown to be suitable for estimation of CoP during movements. Although the difference of RMS error of CoP between the three-link and the five-link models was about 1 cm, the 1 cm difference of the CoP is estimated to cause a change in the ankle joint moment of approximately 0.05 Nm/kg. Although improving estimation error of inclination angles decreases estimation error of ground reaction force and CoP, movements of markers for a camera-based motion measurement system also increase the estimation error by varying reference value. Since inertial sensor-based method achieved high correlation coefficients, the SI method is suggested to be an option for estimating information of ground reaction force.

A low pass filter for estimated or measured angles was used in order to decrease high frequency noise in CoM accelerations caused by calculating the 2nd-order differential. The cutoff frequency of the low pass filter for acceleration signals in Kalman filtering were also determined considering the high frequency noise caused by calculation of the 2nd-order differential after verification of angle estimation with different cutoff frequencies, in which the estimation errors were almost the same between 1.0 Hz and 10 Hz cutoff frequencies. Both filtering did not affect estimation of inclination angles. As another filter, a zero-phase Butterworth low pass filter was tested for CoM acceleration, and it was considered that the cutoff frequency would be dependent on movements. Cutoff frequency of 2 Hz was appropriate for movements measured in this paper. 

### 4.3. Joint Moment Estimation Method for an Easy to Use System

Estimation accuracy of the sensor-based top-down methods (the SI and the SA methods) was similar to the error of the camera-based top-down method (CI method). This suggests that inertial sensors can be used as an alternative to a camera-based motion measurement system in the top-down method. The CI method has the limitation that movements are measured only within the photography range of the 3D motion measurement system. The SA method requires that the inertial sensors be mounted on the CoM position of each segment. The SI method is considered to be easier to use in clinical applications as it has no such limitations. 

Increasing the number of segments for the trunk modeling improves estimation accuracy of joint moments. However, the increase of the segment number decreases the usefulness of joint moment estimation with inertial sensors. Since there were no significant differences in estimation accuracy between the four-link-IC and the five-link models, the four-link-IC model can be used for trunk modeling. The four-link-IC model was considered to be more appropriate than the four-link-R model because the lower trunk moved differently from the middle trunk (Figure 5). As shown in Figure 7, RMS values and correlation coefficients for the four-link-IC model were not different using different sensors, s1 or s2. This suggests that the four-link-IC model can be applied easily, reducing the limitation of sensor location for measurement of movements of the upper-middle trunk segment. For example, sensors for the thigh, the shank and the upper-middle segments can be attached easily by stretchable bands [3]. An attachment method for sensor s3 has to be further studied for practical use.

The SI method with four-link-IC model is considered to be useful as an easy to use system. In this case, RMS values were about 0.1 Nm/kg for all the lower limb joints and correlation coefficients were about 0.98 for the hip and the knee joints and about 0.80 for the ankle joint on average. These values were similar to those of the camera-based top-down method (CI method). Estimation error of the CI method shows differences in estimated joint moments using camera-based motion measurement system between bottom-up inverse dynamic analysis method with force plate and top-down analysis method without force plate. The differences are considered to depend on estimation accuracy of body segment inertial parameter values for each subject [28]. Since joint moments obtained by the conventional method are also estimated values, joint moments estimated by the same estimation method with same measurement system should be compared in applications. From this point of view, the sensor-based estimation method is considered to be used properly because the method had a high correlation with the conventional method.

Joint moment estimation by inertial sensors and force plate showed RMS values of about 0.06 Nm/kg, which is similar to the average absolute difference reported by Faber et al. [25]. Correlation coefficients were larger than 0.98. It would be possible to use inertial sensors with the bottom-up estimation method with shoes that measure ground reaction forces with satisfactorily high accuracy. This can be an option for practical use of joint moment estimation with inertial sensors.

In this paper, joint moment estimation methods were only examined for sit-to-stand and squat movements in the sagittal plane. This is because joint moments in the sagittal plane provide useful information when evaluating muscle strength, various diseases, paralysis, and so on. However, further evaluations of joint moment estimation during various movements such as walking may be necessary for practical application of inertial sensor-based method, because results of this paper were obtained using models that assumed each segment moves in the sagittal plane symmetrically, and feet were fixed on the ground. In addition, joint moments in the frontal plane are needed in order to realize 3D joint moment analysis for further applications. The segmented trunk models have to be evaluated in estimation of joint moments during movements in the frontal plane and also during three-dimensional movements. Since each trunk segment is not rigid, a method using a small number of inertial sensors with four-link or five-link models has a limited ability to estimate joint moment of trunk nodes. A wearable system using a large number of inertial sensors may be suitable for more accurate estimation. The simplified joint moment estimation method of trunk nodes using inertial sensors with four-link model may be an option when other methods cannot be used.

## 5. Conclusions

This paper aimed at quantitative evaluation of inertial sensor-based estimation methods of joint moments and ground reaction forces in the sagittal plane with three-link, four-link and five-link rigid body models that consisted of different trunk segmented models. Lower limb joint moments were estimated for sit-to-stand and squat movements using top-down inverse dynamics analysis and compared to those estimated using the bottom-up analysis method with a camera-based motion measurement system and a force plate. The five-link model and the four-link-IC model, when the trunk was divided at the highest point of the iliac crest, were found to be appropriate for estimation of lower limb joint moments, in which RMS values were about 0.1 Nm/kg for all lower limb joints and correlation coefficients were on average about 0.98 for hip and knee joints and about 0.80 for ankle joint. There were no large differences in estimation results by the top-down methods between using inertial sensors and using camera-based motion measurement system. In addition, the inertial sensor-based method was suggested to be an option for estimating joint moments of the link nodes of the trunk segment, ground reaction forces and CoP with the five-link and the four-link-IC models. Average RMS errors of horizontal and vertical GRFs and CoP were about 10 N, 15 N and 2 cm, respectively. Inertial sensors were shown to be useful for the bottom-up estimation method of joint moments using measured ground reaction forces, which showed the results that average RMS values and average correlation coefficients were about 0.06 Nm/kg and larger than about 0.98 for all joints, respectively. It was considered that the SI method with the four-link-IC model would be easy to use for practical applications of inertial sensor-based joint moment estimation. Inertial sensors are expected to be used as an easy to use system to estimate joint moments, ground reaction force and CoP.

## Figures and Tables

**Table 1:** Calculated values of body segment parameters. Ratios of segment length (%height), segment mass (%weight), and center of mass location from the distal end and radius of gyration (%segment length) are shown.

Segment	Segment Length	Mass	Center of Mass Location	Radius of Gyration
foot	14.8	1.1	0.595	17.7
shank	23.0	5.1	0.594	27.4
thigh	23.9	11.0	0.525	27.8
lower trunk	8.1	15.4	0.397	52.5
middle trunk	3.0	3.3	0.500	97.4
upper trunk	37.8	46.9	0.331	26.9
middle-lower	11.1	18.7	0.391	42.5
upper-middle	40.8	50.2	0.358	25.6
HAT	48.9	65.6	0.371	25.5

**Table 2:** RMS errors and correlation coefficients of segment inclination angles between estimation with inertial sensors and measurement with markers mounted on their sensors by the rigid bar. s1, s2 and s3 are the sensors mounted on the trunk shown in Figure 3a.

Segment	Position	RMSE (°)	CC
thigh	frontal	2.33 ± 0.69	1.00 ± 0.00
	lateral	2.34 ± 0.80	1.00 ± 0.00
shank	frontal	0.60 ± 0.33	1.00 ± 0.00
	lateral	0.59 ± 0.31	1.00 ± 0.00
trunk	s1	1.39 ± 0.88	1.00 ± 0.00
	s2	0.94 ± 0.52	1.00 ± 0.00
	s3	1.02 ± 0.70	0.98 ± 0.08
